# TREM2/DAP12 Complex Regulates Inflammatory Responses in Microglia via the JNK Signaling Pathway

**DOI:** 10.3389/fnagi.2017.00204

**Published:** 2017-06-21

**Authors:** Li Zhong, Zhen-Lian Zhang, Xinxiu Li, Chunyan Liao, Pengfei Mou, Tingting Wang, Zongqi Wang, Zhe Wang, Min Wei, Huaxi Xu, Guojun Bu, Xiao-Fen Chen

**Affiliations:** ^1^Fujian Provincial Key Laboratory of Neurodegenerative Disease and Aging Research, Institute of Neuroscience, Medical College, Xiamen UniversityXiamen, China; ^2^Neuroscience and Aging Research Center, Sanford-Burnham-Prebys Medical Discovery InstituteLa Jolla, CA, United States; ^3^Department of Neuroscience, Mayo ClinicJacksonville, FL, United States; ^4^Shenzhen Research Institute of Xiamen UniversityShenzhen, China

**Keywords:** DAP12, TREM2, JNK, LPS, microglia, inflammation

## Abstract

DNAX-activating protein of 12 kDa (DAP12) is a signaling adapter protein expressed in cells that participate in innate immune responses. By pairing with different triggering receptors expressed on myeloid cell (TREM) proteins, DAP12 can mediate both positive and negative cellular responses. In particular, TREM1 acts as an amplifier of the immune response, while TREM2 functions as a negative regulator. TREM2 has also been shown to stimulate the phagocytosis of apoptotic neurons and define the barrier function in microglia. Notably, loss-of-function mutations of either *DAP12* or *TREM2* result in a disorder known as Nasu-Hakola disease (NHD); and mutations of these genes have been associated with the risk for Alzheimer’s disease (AD), suggesting that TREM2 and DAP12 may regulate common signaling pathways in the disease pathogenesis. In this study, we demonstrated an anti-inflammatory role of DAP12 in murine microglia that depends on the presence of TREM2. We also uncovered the JNK signaling pathway as the underlying molecular mechanism by which the TREM2/DAP12 complex suppresses the hyperactivation of microglia upon LPS stimulation. Interestingly, LPS down-regulates the expression of *Trem2* via the activation of JNK and NF-κB signaling pathways, resulting in a vicious cycle that synergistically promotes the inflammatory responses. Our study provides insights into mechanism-based therapy for neuroinflammatory disorders.

## Introduction

DNAX-activating protein of 12 kDa (DAP12; also known as TYROBP and KARAP) is a signaling adapter protein expressed by a variety of innate immune cells including macrophages, microglia, monocytes, dendritic cells and natural killer (NK) cells (Lanier, 2009). The broad distribution of DAP12 suggests a general function in immune responses. DAP12 consists of a minimal extracellular domain, a transmembrane segment and a cytoplasmic region containing a single immunoreceptor tyrosine-based activation motif (ITAM). An aspartic acid in the transmembrane domain of DAP12 allows its association with cell surface receptors via an electrostatic interaction. The receptors usually have an oppositely charged amino acid (arginine or lysine) embedded within their transmembrane region that allows the formation of non-covalent complexes with DAP12 (Lanier and Bakker, 2000; Humphrey et al., 2005). Ligation of a DAP12-associated receptor to its ligand leads to the activation of SRC-family kinases and subsequent phosphorylation of tyrosine residues in the ITAM of DAP12 (Mason et al., 2006). DAP12 was originally shown to trigger the activation of NK cells (Lanier et al., 1998). Since then, more than 20 DAP12-associated receptors have been identified (Turnbull and Colonna, 2007). Triggering receptors expressed on myeloid cells (TREMs) are a family of cell surface receptors expressed broadly on myeloid cells that have been identified to associate with DAP12 (Bouchon et al., 2000; Daws et al., 2001; Chung et al., 2002). In particular, TREM1 is a potent amplifier of the inflammatory responses; while TREM2 has an anti-inflammatory function (Bouchon et al., 2000; Gibot et al., 2004; Takahashi et al., 2005; Turnbull et al., 2006).

TREM2 is a DAP12-coupled receptor that acts as a sensor for a wide array of lipids and apolipoprotein E (ApoE) in the central nervous system (CNS; Atagi et al., 2015; Bailey et al., 2015; Wang et al., 2015; Yeh et al., 2016). Notably, loss-of-function mutations of either *DAP12* or *TREM2* result in a disorder known as Nasu-Hakola disease (NHD; Paloneva et al., 2000, 2002). Furthermore, both *TREM2* (Guerreiro et al., 2013; Jonsson et al., 2013) and *DAP12* (Pottier et al., 2016) mutations have been found to be associated with the risk for Alzheimer’s disease (AD). These observations suggest that TREM2 and DAP12 may regulate common signaling pathways in the disease pathogenesis. TREM2 and DAP12 are both preferentially expressed in microglia within the CNS (Sessa et al., 2004). Together, they regulate functions in microglia including inhibition of pro-inflammatory responses and stimulation of phagocytosis of apoptotic neurons (Takahashi et al., 2005; Hamerman et al., 2006; Turnbull et al., 2006; Zhong et al., 2015). Recently, TREM2/DAP12 complex has also been demonstrated to regulate the barrier function in microglia that prevents the outward extension of amyloid fibrils and axonal dystrophy (Sirkis et al., 2016; Yuan et al., 2016).

Despite intense interest in the function of TREM2/DAP12 complex in microglia, current understanding of the relevant molecular, cellular and biophysical mechanisms is limited. Studies elucidating such mechanisms may uncover targetable pathways for AD therapy. In this study, we demonstrated an anti-inflammatory role of DAP12 in murine microglia that requires the function of TREM2. Mechanistically, TREM2/DAP12 suppressed the hyperactivation of JNK signaling pathway upon LPS stimulation. Consequently, a JNK inhibitor, SP600125, eliminated the hypersensitivity of *Dap12*-deficient microglia to LPS. Together, our data suggest that TREM2/DAP12 complex negatively regulates LPS-induced inflammatory responses by modulating the JNK signaling pathway in microglia.

## Materials and Methods

### Reagents and Antibodies

Amaxa^®^ Cell Line Nucleofector^®^ Kit T and Amaxa^®^ Glia Cell Nucleofector^®^ Kit T were purchased from LONZA. Primers for quantitative RT-PCR were synthesized by Life Technologies. SYBR Green for quantitative RT-PCR was purchased from Roche. SP600125, Bay11–7082, SB203580, U0126 and LPS were purchased from Sigma. Amyloid-β 42 (Aβ42) peptide was purchased from AnaSpec. Oligomeric Aβ42 was prepared as previously described (Huang et al., 2015). Antibodies used in this study are as followed: anti-phospho-p38-MAPK, anti-total-p38-MAPK, anti-phospho-ERK1/2, anti-total-ERK1/2, anti-phospho-JNK, anti-total-JNK, anti-phospho-IκBα, anti-total-IκBα, anti-phosho-NF-κB, anti-total-NF-κB, anti-phospho-c-Jun, anti-total-c-Jun and anti-β-actin were purchased from Cell Signaling Technology; anti-tubulin (Millipore); anti-mouse IgG and anti-rabbit IgG antibody conjugated with horseradish peroxidase (ThermoFisher Scientific).

### Isolation and Culture of Mouse Primary Microglia

*Trem2* knockout mice (*Trem2*-KO on C57BL/6N background) and wild-type (WT) C57BL/6N mice were obtained from the UC Davis Knockout Mouse Project (KOMP) repository. The exons 2–4 of the *Trem2* gene were replaced with a LacZ reporter which is identical to the line recently reported (Jay et al., 2015). Primary microglial cultures were prepared as previously described (Zhu et al., 2010; Atagi et al., 2015). All animal experiments were conducted in compliance with the protocols approved by the Institutional Animal Care and Use Committee of Xiamen University. Briefly, WT or *Trem2*-KO mice (3–4 pups) at postnatal day 1–2 were used to prepare mixed glial cultures. Cells were plated onto flasks and grown in DMEM supplemented with 10% heat-inactivated fetal bovine serum (FBS; Gibco). Three days later, medium was changed to that containing 25 ng/mL GM-CSF and 10% FBS. Primary microglia were harvested by shaking (200 rpm, 20 min) after 10–12 days in culture and once every 3 days thereafter (up to three harvests).

### Western Blotting

BV2 microglial cells or primary microglia were lysed at the indicated times with lysis buffer (1% NP-40, 50 mM Tris-HCl, pH 8.0, 150 mM sodium chloride) supplemented with protease and phosphotase inhibitor cocktails. BCA protein assay kit was used to determine the protein concentration according to the manufacturer’s instruction (ThermoFisher Scientific). Equal amounts of total proteins were analyzed by sodium dodecyl sulfate-polyacrylamide gel electrophoresis (SDS-PAGE) and Western blotting using appropriate antibodies and HRP-conjugated secondary antibodies. Proteins were visualized using ECL Western blotting detection reagents (Millipore). Immunoreactive bands were quantified using ImageJ.

### Quantitative RT-PCR

Total RNAs were extracted using TRIzol reagent (Invitrogen). One microgram RNA was reverse-transcribed into first-strand cDNA using TransScript All-in-One First-Strand cDNA Synthesis SuperMix (TRANSGEN BIOTECH, Beijing, China) according to the manufacturer’s protocol. Quantitative PCR was performed using the FastStart Universal SYBR Green Master (Roche). The primer sequences used for Dap12, Trem2, IL-1β, TNF-α, IL-6 and β-Actin were the same as previously described (Zhong et al., 2015, 2017).

### RNA Interference

siRNA at a concentration of 300 nM was transfected into BV2 cells using Amaxa^®^ Cell Line Nucleofector^®^ Kit T or primary microglia cells using Amaxa^®^ Glia Cell Nucleofector^®^ Kit T. Cells were harvested 48 h later, followed by RNA extraction for quantitative RT-PCR analysis or protein extraction for Western blotting analysis. The siRNA sequences for Dap12 were the same as previously described (Zhong et al., 2015).

### Statistical Analyses

Statistical analyses were performed using GraphPad Prism and all data were presented as mean ± SEM. At least three independent experiments were analyzed by unpaired *t*-test, one-way ANOVA or two-way ANOVA test. To classify and indicate significant values, the following *p*-values were used: *p < 0.05; **p < 0.01; ***p < 0.001; ns, not significant.

## Results

### DAP2 Inhibits LPS-Induced Cytokines Production Dependent on TREM2 Receptor

**Figure 1:**
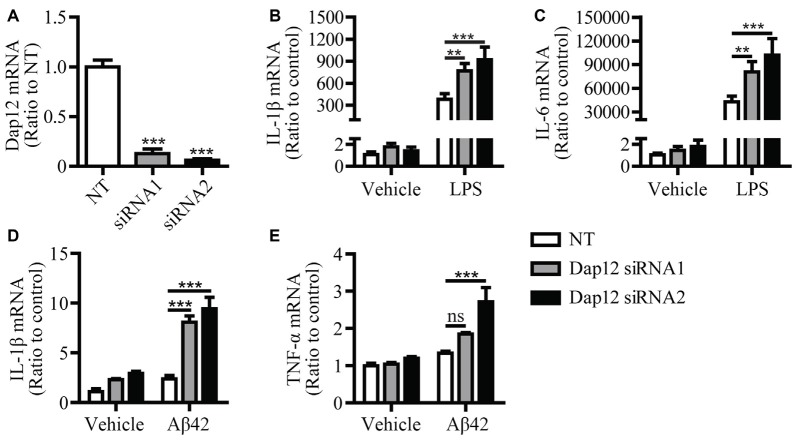
Knockdown of *Dap12* exacerbates LPS- or Aβ42-oligomer-stimulated production of pro-inflammatory cytokines. **(A)** Primary microglia cells were transiently transfected with non-targeting siRNA (NT) or *Dap12*-specific siRNAs for 48 h. The relative mRNA levels of *Dap12* were determined by quantitative RT-PCR and shown as bar graph (*n* = 3, 1-way ANOVA). **(B,C)** Cells from **(A)** were treated with 500 ng/mL LPS or vehicle control for 4 h. RNA was extracted and the relative mRNA levels of IL-1β **(B)** and IL-6 **(C)** shown as bar graph were determined by quantitative RT-PCR (*n* = 3, two-way ANOVA). **(D–E)** Cells from **(A)** were treated with 10 μM oligomeric-Aβ42 or vehicle control for 4 h. RNA was extracted and the relative mRNA levels of IL-1β and TNF-α shown as bar graph were determined by quantitative RT-PCR (*n* = 3, two-way ANOVA). β-actin was used as an internal control. Data represent mean ± SEM. **p < 0.01; ***p < 0.001; ns, not significant.

In our previous study, we found that knockdown of *Dap12* gene in microglial BV2 cells significantly increased the mRNA levels of pro-inflammatory cytokines in the presence of LPS (Zhong et al., 2015). To further confirm the role of DAP12 in mediating the inflammatory responses to pathogenic stimuli, we employed two *Dap12*-specific siRNAs to knockdown the expression of *Dap12* in primary microglia and examined its impacts on cytokine expression (Figure 1A). Consistently, the knockdown of *Dap12* significantly increased the mRNA levels of IL-1β and IL-6 in LPS-stimulated primary microglia (Figures 1B,C). The production of IL-1β and TNF-α were also elevated in response to treatment with Aβ42 oligomers in *Dap12*-knockdown primary microglia (Figures 1D,E). These data suggest that DAP12 is essential for suppressing the production of pro-inflammatory cytokines when microglial cells are exposed to pathogenic stimuli.

**Figure 2:**
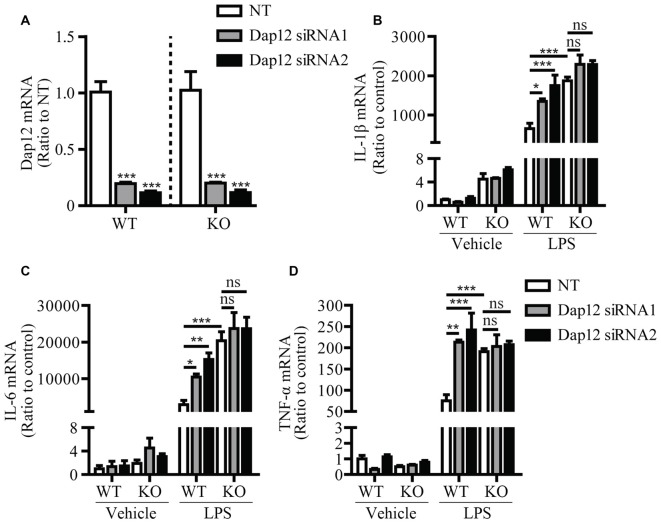
DAP12 regulates inflammatory responses in a manner that depends on the presence of TREM2. **(A)** Primary microglia cells from wild-type (WT) or *Trem2*- knockout (KO) mice were transiently transfected with NT or *Dap12*-specific siRNAs for 48 h. The relative mRNA levels of *Dap12* were determined by quantitative RT-PCR and shown as bar graph (*n* = 3, one-way ANOVA). **(B–D)** Cells from **(A)** were treated with 100 ng/mL LPS or vehicle control for 4 h. RNA was extracted and the relative mRNA levels of IL-1β **(B)** IL-6 **(C)** TNF-α **(D)** shown as bar graph were determined by quantitative RT-PCR (*n* = 3, two-way ANOVA). β-actin was used as an internal control. Data represent mean ± SEM. *p < 0.05; **p < 0.01; ***p < 0.001; ns, not significant.

In cells of myeloid origin, TREM1 and TREM2 are two receptors that signal through DAP12 to oppositely regulate the inflammatory response. TREM1 has been shown to function as an amplifier of the inflammatory response (Bouchon et al., 2000), whereas TREM2 has an anti-inflammatory function (Turnbull et al., 2006). Since we observed an anti-inflammation function of DAP12 in microglia, we further investigated whether DAP12 suppresses the production of inflammatory cytokines in a manner that depends on TREM2. Primary microglia were isolated from both WT and *Trem2*-knockout (KO) mice and further subjected to siRNA treatment that specifically knock down the expression of *Dap1*2 (Figure 2A). Although the deficiency of *Dap1*2 significantly enhanced the production of inflammatory cytokines IL-1β, IL-6 and TNF-α in LPS-stimulated WT primary microglia, the effects were abolished in *Trem2*-KO microglia (Figures 2B–D). It is noteworthy that the amounts of these inflammatory cytokines were significantly higher in *Trem2*-KO microglia than WT microglia, which is consistent with our previous reports (Zheng et al., 2016). Taken together, we conclude that DAP12 suppresses the production of pro-inflammatory cytokines in microglia in a manner that depends on TREM2 receptor.

### Enhanced JNK Phosphorylation in *Trem2*- and *Dap12*-Deficient Microglia

**Figure 3:**
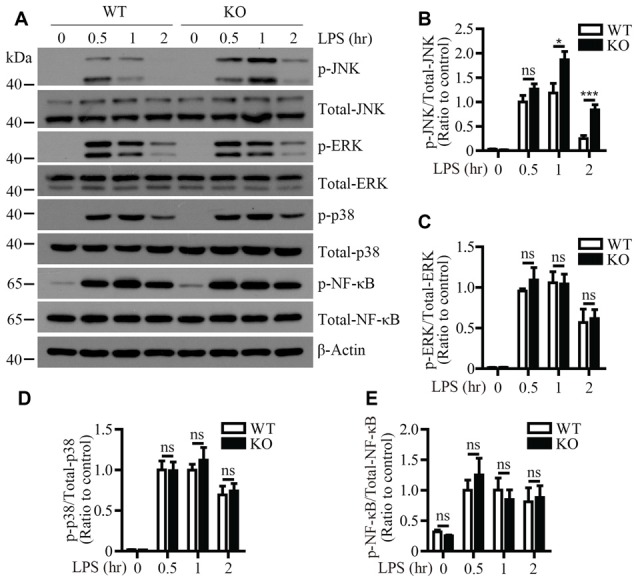
LPS-induced JNK phosphorylation is increased in *Trem2*-deficient microglia. **(A)** Primary microglia cells from WT or *Trem2*- knockout (KO) mice were stimulated with 100 ng/ml LPS for the indicated time. Cell lysates at each time point were analyzed by Western blotting using antibodies specific for either total proteins or phosphorylated form of JNK, P38-MAPK, ERK1/2 and NF-κB. **(B–E)** Bar graphs show the quantification of Western blots as ratios of phospho-JNK/total JNK **(B)** phospho-ERK1/2/total ERK1/2 **(C)** phospho-p38-MAPK/total p38-MAPK **(D)** and phospho-NF-κB/total NF-κB **(E)**, respectively. β-actin was used as an internal control. The ratio at “0.5 h” time point of WT cells served as a control (*n* ≥ 3, unpaired Student’s *t*-test). Data represent mean ± SEM. *p < 0.05; ***p < 0.001; ns, not significant.

**Figure 4:**
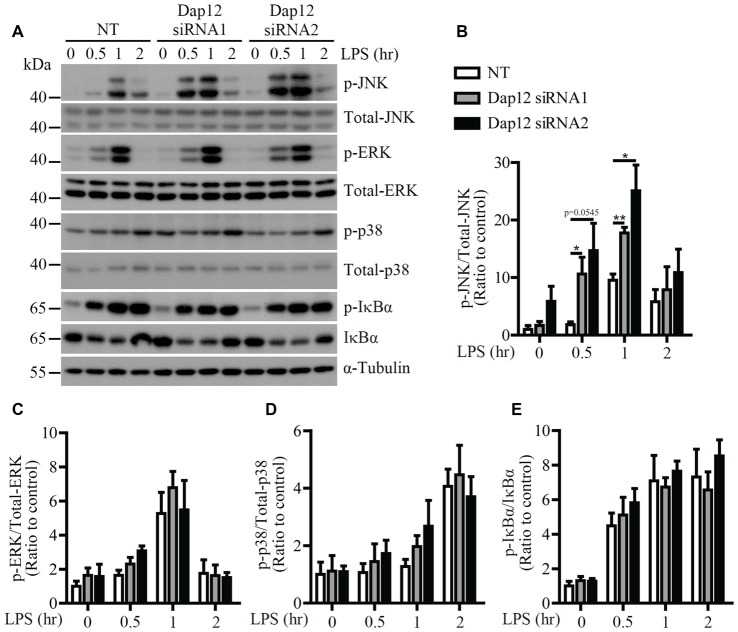
LPS-induced JNK phosphorylation is increased in *Dap12*-knockdown BV2 cells. **(A)** BV2 cells were transiently transfected with NT or *Dap12*-specific siRNAs for 48 h, then stimulated with 500 ng/mL LPS for the indicated times. **(B–E)** Bar graphs show the quantification of Western blots as ratios of phospho-JNK/total JNK **(B)** phospho-ERK1/2/total ERK1/2 **(C)** phospho-p38-MAPK/total p38-MAPK **(D)** and phospho-IκBα/total IκBα **(E)**, respectively. α-Tubulin was used as an internal control. The ratio at “0” time point of NT cells served as a control (*n* = 3, unpaired Student’s *t*-test). Data represent mean ± SEM. *p < 0.05; **p < 0.01.

To identify the signaling pathway(s) that mediates the inflammatory responses induced by LPS in the absence of *Trem2*/*Dap12*, we first examined the activation kinetics of the transcription factor NF-κB and the major MAPKs subtypes (ERK1/2, p38-MAPK and JNK) in both WT and *Trem2*-KO primary microglia. The activation kinetics and magnitude of phosphorylated ERK1/2, p38-MAPK and NF-κB were similar in LPS-stimulated WT and *Trem2*-KO primary microglia (Figures 3A,C–E). In contrast, the phosphorylation of JNK was more pronounced in primary microglia from *Trem2*-KO mice compared with WT mice (Figures 3A,B). Similarly, knockdown of *Dap12* in microglial BV2 cells significantly increased the phosphorylation of JNK (Figures 4A,B), whereas no effects were observed for phosphorylated ERK1/2, p38-MAPK and IκBα, a key regulator in the NF-κB signaling pathway (Figures 4C–E). We therefore conclude that the TREM2/DAP12 complex regulates the inflammatory responses in microglia by specifically blocking the activation of JNK signaling pathway.

### JNK Inhibitor Eliminates the Hypersensitivity of *Dap12*-Deficient Microglia to LPS

**Figure 5:**
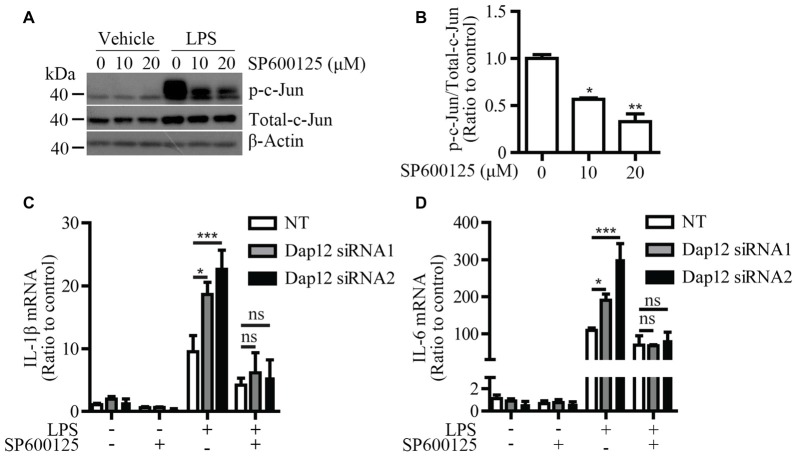
JNK inhibitor reverses the increased pro-inflammatory cytokines in *Dap12*-knockdown BV2 cells. **(A)** BV2 cells were pretreated with indicated concentrations of SP600125 for 30 min, and then stimulated with 500 ng/mL LPS or vehicle control for 1 h. Cell lysates were analyzed by Western blotting. **(B)** Bar graphs show the quantification of Western blots as ratios of phospho-c-Jun/total c-Jun (*n* = 3, one-way ANOVA). **(C,D)** BV2 cells were transiently transfected with non-targeting siRNA (NT) or *Dap12*-specific siRNAs for 48 h, and then stimulated with 500 ng/mL LPS or vehicle control for 4 h in the presence or absence of 20 μM SP600125 (pretreated for 30 min). RNA was extracted and the relative mRNA levels of IL-1β and IL-6 shown as bar graph were determined by quantitative RT-PCR (*n* = 3, two-way ANOVA). β-actin was used as an internal control. *p < 0.05; **p < 0.01; ***p < 0.001; ns, not significant.

To further explore the molecular mechanism by which *Dap12* down-regulation affects the pro-inflammatory responses induced by LPS, a specific inhibitor (SP600125) was used to block the activation of JNK signaling pathway. Microglial BV2 cells were pre-treated with SP600125 before LPS stimulation. At a dose of 10 or 20 μM, SP600125 inhibits the phosphorylation of c-Jun which is a downstream target of JNK pathway (Figures 5A,B). The mRNA levels of pro-inflammatory cytokines IL-1β and IL-6 were increased upon knockdown of *Dap12*; however, the effect was abolished by pre-treatment with the JNK inhibitor (Figures 5C,D). Taken together, these data indicated that DAP12 negatively regulates LPS-induced inflammatory responses in microglia by modulating the activity of JNK signaling pathway.

### LPS-Induced Down-Regulation of *Trem2* is Rescued by JNK and NF-κB Inhibitors

**Figure 6:**
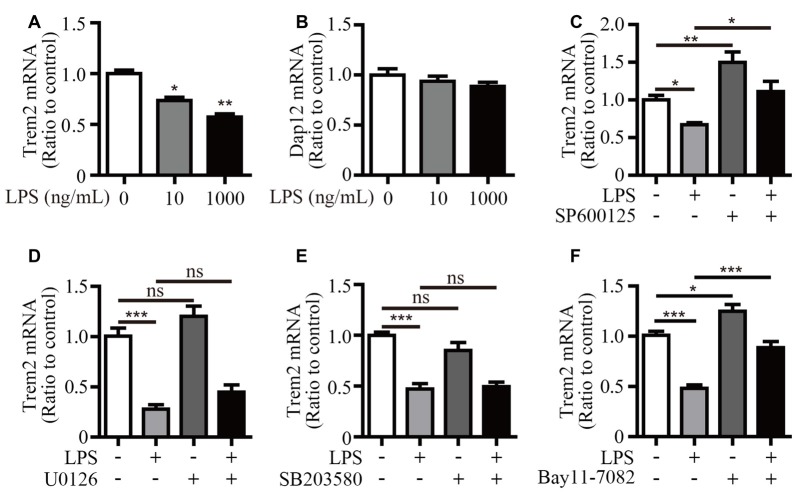
Inhibitors for JNK and NF-κB restore *Trem2* expression suppressed by LPS. **(A,B)** BV2 cells were treated with indicated concentrations of LPS (0, 10 and 1000 ng/mL) for 4 h. RNA was extracted and the relative mRNA levels of *Trem2* **(A)** or *Dap12* **(B)** shown as bar graph was determined by quantitative RT-PCR (*n* = 3, one-way ANOVA). **(C–F)** BV2 cells were pretreated with 10 μM SP600125 **(C)**, 5 μM U0126 **(D)**, 5 μM SB203580 **(E)** or 5 μM Bay11–7082 **(F)** for 30 min, followed by treatment with 500 ng/mL LPS or vehicle control for 12 h. RNA was extracted and the relative mRNA levels of *Trem2* shown as bar graph were determined by quantitative RT-PCR (*n* ≥ 3, one-way ANOVA). β-actin was used as an internal control. *p < 0.05; **p < 0.01; ***p < 0.001; ns, not significant.

We have previously shown that LPS stimulation significantly suppressed *Trem2* expression in primary microglia and mouse brain (Zheng et al., 2016). Consistently, the mRNA levels of *Trem2* were significantly down-regulated in LPS stimulated microglial BV2 cells (Figure 6A). However, the expression of *Dap12* was unaffected even upon the stimulation with 1 μg/mL LPS (Figure 6B). To further dissect the molecular pathway that modulates *Trem2* expression, we pretreated BV2 cells with various compounds that specifically block individual signaling pathways downstream of LPS, including NF-κB and each of the major MAP kinase subtypes. The mRNA level of *Trem2* was similarly down-regulated by LPS in the presence or absence of p38-MAPK and ERK1/2 inhibitors (Figures 6D,E). However, the LPS down-regulated *Trem2* expression was restored by the presence of inhibitors for either JNK or NF-κB (Figures 6C,F). Taken together, our data suggest that both JNK and NF-κB signaling pathways downstream of LPS modulate the expression of *Trem2* in microglia.

## Discussion

**Figure 7:**
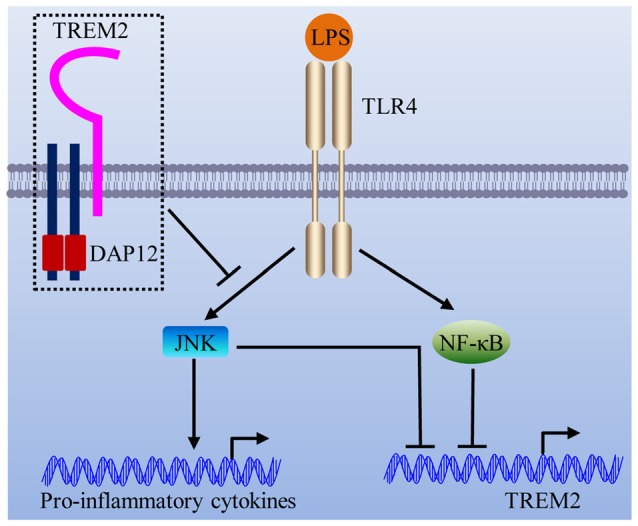
Schematic model of the interplay between TREM2/DAP12 complex and LPS-induced inflammation. The TREM2/DAP12 complex suppresses the activity of JNK signaling pathway to reduce LPS-triggered inflammatory response in microglia. LPS down-regulates the expression of *Trem2* via the activation of JNK and NF-κB signaling pathways.

In this study, we showed that DAP12 suppresses the production of pro-inflammatory cytokines when microglial cells are exposed to LPS. Importantly, the negative modulation of inflammatory response by DAP12 depends on the presence of TREM2. In view of the underlying molecular mechanism, we revealed that the TREM2/DAP12 axis suppresses the activity of JNK signaling pathway to reduce the inflammatory response in microglia (Figure 7). Intriguingly, LPS down-regulates the expression of *Trem2* via the activation of JNK and NF-κB signaling pathways (Figure 7), resulting in a vicious cycle that synergistically promotes the inflammatory responses.

As a signaling adaptor protein, DAP12 couples with a variety of cell-surface receptors to modulate the threshold for cellular activation in response to pathogenic stimuli (Turnbull and Colonna, 2007). For instance, the association of DAP12 with TREM1 has been shown to amplify the inflammatory response (Bouchon et al., 2000; Gibot et al., 2004); while the association with TREM2 dampens the production of pro-inflammatory cytokines (Takahashi et al., 2005; Turnbull et al., 2006). Therefore, the roles of DAP12 in different cell types could be varied depending on the presence of specific cell surface receptors. DAP12 is preferentially expressed in microglia within the CNS (Hickman et al., 2013). In our previous work and in current study, we have demonstrated that DAP12 inhibits the production of pro-inflammatory cytokines in LPS-stimulated microglia by using both immortalized cell line and primary cultures (Zhong et al., 2015). We further demonstrated that DAP12 exerts its anti-inflammatory function by coupling with TREM2 which is the highest expressed DAP12-associated receptor in microglia among TREM family members (Zhang et al., 2014). Interestingly, the DAP12 signaling has been shown to amplify inflammation during sepsis (Turnbull et al., 2005). The receptors that are involved remain unknown; TREM1, for instance, might be needed for DAP12 to signal in a pro-inflammatory manner. The activating and inhibitory functions of DAP12 in inflammation are proposed to be modulated by the avidity of the interaction between the DAP12-associated receptor and its ligand (Turnbull and Colonna, 2007).

In accordance with our findings in microglia, *Dap12*-deficient macrophages have been reported to express higher amounts of inflammatory cytokines in response to a variety of pathogenic stimuli (Hamerman et al., 2005). However, the signaling mechanism by which Dap12 regulates cytokine production was distinct between microglia and macrophages. Upon LPS stimulation, ERK1/2 signaling was more pronounced in *Dap12*-deficient macrophages than in WT cells (Hamerman et al., 2005). In contrast, we observed the activation of JNK signaling pathway in* Trem2*- and *Dap12*-deficient microglia. It remains uncharacterized how DAP12 regulates the phosphorylation of either ERK1/2 in macrophages or JNK in microglia. Further study is needed to define the precise molecular pathway downstream of DAP12 actions.

We and others have consistently shown that LPS stimulation significantly suppressed microglial *Trem2* expression both *in vitro* and *in vivo* (Schmid et al., 2002; Zheng et al., 2016). The decrease in *Trem2* expression further augments the production of inflammatory cytokines, leading to detrimental exaggeration of neuroinflammation (Zhong et al., 2015; Zheng et al., 2016). Therefore, understanding the molecular mechanism by which LPS or other pathogenic stimuli regulate *Trem2* expression would provide insights into eliminating the source of inflammation cascade. Our current study showed that applying either JNK or NF-κB inhibitor restored *Trem2* expression down-regulated by LPS, implicating a potentially beneficial effect of those inhibitors for treating neurological diseases with an inflammatory component. The precise molecular pathways downstream of JNK and NF-κB require further investigation. It would be intriguing to examine whether the transcription factors activated by JNK and NF-κB regulate *Trem2* expression via direct binding to its proximal promoter.

Collectively, our studies revealed that DAP12 possesses an anti-inflammatory function in murine microglia that is TREM2-dependent. The TREM2/DAP12 axis negatively regulates the activity of JNK signaling pathway downstream of LPS to suppress the inflammatory responses. Our study provides insights into mechanism-based therapy for neuroinflammatory disorders.

## Author Contributions

LZ, X-FC and GB: designed research; LZ, Z-LZ, XL, CL, PM, TW, ZQW, ZW and MW: performed experiments; LZ, Z-LZ, XL, CL and X-FC: analyzed data; X-FC and LZ: wrote the manuscript; HX and GB: reviewed the manuscript. All authors read and approved the final manuscript.

## Conflict of Interest Statement

The authors declare that the research was conducted in the absence of any commercial or financial relationships that could be construed as a potential conflict of interest.
